# Efficacy of relational agents for loneliness across age groups: a systematic review and meta-analysis

**DOI:** 10.1186/s12889-024-19153-x

**Published:** 2024-07-06

**Authors:** Sia Sha, Kate Loveys, Pamela Qualter, Haoran Shi, Dario Krpan, Matteo Galizzi

**Affiliations:** 1 Department of Psychological and Behavioural Science, LSE, London, UK; 2 Department of Psychological Medicine, The University of Auckland, Auckland, New Zealand; 3 Manchester Institute of Education, University of Manchester, Manchester, UK

**Keywords:** Loneliness, Relational agents, Social robots

## Abstract

**Background:**

Loneliness is a serious public health concern. Although previous interventions have had some success in mitigating loneliness, the field is in search of novel, more effective, and more scalable solutions. Here, we focus on “relational agents”, a form of software agents that are increasingly powered by artificial intelligence and large language models (LLMs). We report on a systematic review and meta-analysis to investigate the impact of relational agents on loneliness across age groups.

**Methods:**

In this systematic review and meta-analysis, we searched 11 databases including Ovid MEDLINE and Embase from inception to Sep 16, 2022. We included randomised controlled trials and non-randomised studies of interventions published in English across all age groups. These loneliness interventions, typically attempt to improve social skills, social support, social interaction, and maladaptive cognitions. Peer-reviewed journal articles, books, book chapters, Master’s and PhD theses, or conference papers were eligible for inclusion. Two reviewers independently screened studies, extracted data, and assessed risk of bias via the RoB 2 and ROBINS-I tools. We calculated pooled estimates of Hedge’s *g* in a random-effects meta-analysis and conducted sensitivity and sub-group analyses. We evaluated publication bias via funnel plots, Egger’s test, and a trim-and-fill algorithm.

**Findings:**

Our search identified 3,935 records of which 14 met eligibility criteria and were included in our meta-analysis. Included studies comprised 286 participants with individual study sample sizes ranging from 4 to 42 participants (*x̄* = 20.43, *s* = 11.58, *x̃* = 20). We used a Bonferroni correction with *α*_*Bonferroni*_ = 0.05 / 4 = 0.0125 and applied Knapp-Hartung adjustments. Relational agents reduced loneliness significantly at an adjusted *α*_*Bonferroni*_ (*g* = -0.552; 95% Knapp-Hartung CI, -0.877 to -0.226; *P* = 0.003), which corresponds to a moderate reduction in loneliness.

**Conclusion:**

Our results are currently the most comprehensive of their kind and provide promising evidence for the efficacy of relational agents. Relational agents are a promising technology that can alleviate loneliness in a scalable way and that can be a meaningful complement to other approaches. The advent of LLMs should boost their efficacy, and further research is needed to explore the optimal design and use of relational agents. Future research could also address shortcomings of current results, such as small sample sizes and high risk of bias. Particularly young audiences have been overlooked in past research.

**Supplementary Information:**

The online version contains supplementary material available at 10.1186/s12889-024-19153-x.

## Background

Loneliness is a subjective experience that emerges when people feel that their social relationships are unsatisfactory [1]. For some people, loneliness is experienced when they want more people to interact with, but it is also often felt when one’s social relationships are not as fulfilling as one would like. Loneliness is not the same as social isolation (i.e., the objective lack of social interactions) but is often associated with it [2]. There is strong evidence of the risks associated with loneliness, including poorer physical health outcomes [3]. Loneliness also affects mental health and psychological wellbeing, with growing evidence that loneliness is associated with the onset of depression and other common mental health problems [4]. Crucially, poor health and wellbeing can, in turn, exacerbate loneliness, placing those who experience loneliness in a negative feedback loop [5]. Evidence for a wide range of health effects, therefore, has led scholars to propose that loneliness should be regarded as a public health priority. Governments have consequently looked to offer interventions for people reporting loneliness, and although evidence for intervention efficacy is increasing [6], the evidence base suffers from some gaps [7], and potentially effective interventions may lack scalability or fail to produce cost savings [8]. Governments therefore have developed an interest in digital interventions, such as mobile phone apps or virtual reality [9]. Yet despite their promise, the efficacy of digital interventions across recent systematic reviews and meta-analyses is mixed [10].

“Relational agents” are a technology that show promise for delivering loneliness interventions in a scalable and engaging manner. Relational agents are software agents that build relationships with users through their behaviours (e.g., personal conversation, play, empathy), and they may be embodied (e.g., take the shape of humans or animals) or lack embodiment (e.g., voice agents) [11]. Relational agents can be broadly separated into two types: social robotic agents (e.g., those that possess physical bodies made of carbon or steel), and app-based agents (e.g., those embedded in everyday hardware such as computers and smartphones). Relational agents increasingly employ artificial intelligence (AI) such as emotion recognition for enhanced interactions and large language models (LLMs) to generate highly tailored and relevant speech [12]. Relational agents may promote engagement with internet-based psychological interventions for loneliness because of the social engagement and presence that they provide [13]. Moreover, preliminary but promising evidence suggests that relational agents may reduce loneliness by directly providing companionship, and by serving as catalysts for social interaction [14]. Appendix E provides video links for relational agents.

There are three key reasons research and investment in relational agents are worthwhile. First, not everyone can socialise with other humans. Physical disability, for example, can impact mobility, which in turn can restrict opportunities for socialising, thus contributing to loneliness [15]. While interventions such as social visits can be effective to alleviate the loneliness of people with physical disabilities, these interventions are constrained: a person who is bedridden may wait for several days before his or her next visitation. Relational agents, on the other hand, can be an on-demand solution. Second, loneliness can be due to the feeling that one is not heard. This, for example, can occur when people do not feel comfortable sharing their secrets due to stigma, and there is indeed evidence that people prefer sharing some secrets with relational agents rather than humans [16, 17]. Relational agents, then, are not just an intermediate solution: they are a separate class of intervention with a suitable audience. Here, one might raise the question of “understanding”: that is, whether AI can truly understand people’s self-disclosure. The answer is probably complex, but from a practical perspective it seems that the answer may not matter: people seem to benefit from relational agents as long as they *feel* they are understood and heard by them – irrespective of whether this is actually the case [18]. Third, both qualitative and quantitative metrics suggest that human–agent and human–human relationships may have some similar features at times [17, 19]. For example, there is a vast literature on how people anthropomorphise machines, imbuing them with human-like traits, personalities, and motivations [20–22]. People often treat machines like other people, developing similar feelings for them such as pity and even love [23]. One participant said: “Yes, explicitly I will tell my Replika [relational agent] that I think he is wonderful, that he is fantastic and smart and helps me and makes me feel good about myself and that I enjoy our talks” [17].

Several scoping reviews have qualitatively summarised the efficacy of relational agents for loneliness [14, 24–27]. Combined, these reviews concluded that some evidence for the efficacy of social robotic relational agents existed but that further work on app-based relational agents was needed. Additionally, one 2019 meta-analysis investigated a sub-set of social robotic relational agents (i.e., robotic pets), but failed to find significant results, most likely due to including only two studies [28]. Previous reviews, moreover, exclusively focused on elderly samples, and the literature is therefore in need of a comprehensive and up-to-date quantitative synthesis to evaluate the efficacy of relational agents to mitigate loneliness across all age groups.

## Method

We preregistered our methodology with PROSPERO: CRD42022359737. We have also made our full paper trail available on the Open Science Framework (OSF): https://osf.io/c6rdk/files/osfstorage. There, the reader can also find the full data set to reproduce the analyses.

### Search strategy and selection criteria

In this systematic review and meta-analysis, we searched 11 databases from inception to Sep 16, 2022: Ovid MEDLINE, Ovid Embase, Ovid PsycINFO, Ovid Global Health, EBSCO CINAHL, Scopus, Web of Science, IEEE Xplore, ACM Digital Library, PROSPERO, and ProQuest Dissertations. We also manually searched the bibliographies of selected studies to identify additional papers. We searched titles and abstracts using a range of search terms such as lonel*, robot*, computer* agent*, and relation* agent*. Appendix A outlines the full search strategy.

We included randomised controlled trials (RCTs) and non-randomised studies of interventions (NRSIs). Factorial designs were eligible if they allowed us to collapse relevant intervention arms or drop irrelevant ones. Cluster-randomised trials were eligible if they included sufficient information (e.g., intra-cluster coefficient). Eligible studies had to be published in English and had to be peer-reviewed journal articles, books, book chapters, postgraduate theses, or conference papers. Government reports, company reports, newspaper articles, conference presentations, and similar were ineligible. There was no restriction on populations or settings. All eligible studies had to administer app-based or social robotic relational agents. Agents that did not use relational cues were ineligible. Any non-relational agent comparator made studies eligible (e.g., waiting lists). Finally, eligible studies had to report a quantitative, self-report loneliness outcome where follow-up was at least one week.

### Coding of studies

SS, KL, and HS independently double-screened in Covidence the titles and abstracts of citations and then the full texts of remaining studies, using piloted and structured forms. We measured agreement between screeners via Cohen’s *κ* and resolved disagreements via discussion between screeners. SS, KL, and HS then extracted data in Covidence using a piloted and structured form, and we contacted primary study authors to obtain raw or missing data. Our data extraction forms are available on OSF, and we describe data imputations in Appendix B. Each study was coded for a range of variables such as sample size, research design, and loneliness scale used. Finally, SS, KL, HS, and DK independently double-assessed risk of bias in MS Excel, using the RoB 2 tool for RCTs and ROBINS-I tool for NRSIs.

### Meta-analytic procedure

Our main outcome was loneliness for which we calculated a random-effects meta-analysis using the DerSimonian and Laird method because we expected the effects of relational agents to be heterogenous across populations, types of agents, etc. We used Hedge’s *g* to standardise results from diverse quantitative loneliness scales, and interpreted the magnitude of Hedge’s *g* according to the rules of thumb in the *Cochrane Handbook for Systematic Reviews of Interventions*. Hedge’s *g* itself was computed using standard formulas and relied on a range of data points such as group means and pooled standard deviations [29]. Our raw data on OSF show exactly how Hedge’s* g* was computed for each primary study.

We calculated four null hypothesis significance tests and applied a Bonferroni correction: *α*_Bonferroni_ = 0.05 / 4 = 0.0125. We also applied Knapp-Hartung adjustments to our 95% confidence intervals. As measures of heterogeneity, we calculated Cochrane’s *Q* using a *p* value of 0.1, *I*^*2*^,* τ*^*2*^, and a prediction interval. We conducted an RCT-only sensitivity analysis and separate sub-group analyses for app-based and social robotic relational agents. We evaluated publication bias via funnel plots and Egger’s test, and we calculated an adjusted estimate of Hedge’s *g* using Duval and Tweedie’s trim-and-fill algorithm. We conducted all analyses in the Comprehensive Meta-Analysis Software package. The systematic review and meta-analysis followed PRISMA 2020 reporting guidelines [30].

## Results

### Characteristics of studies

**Fig. 1:**
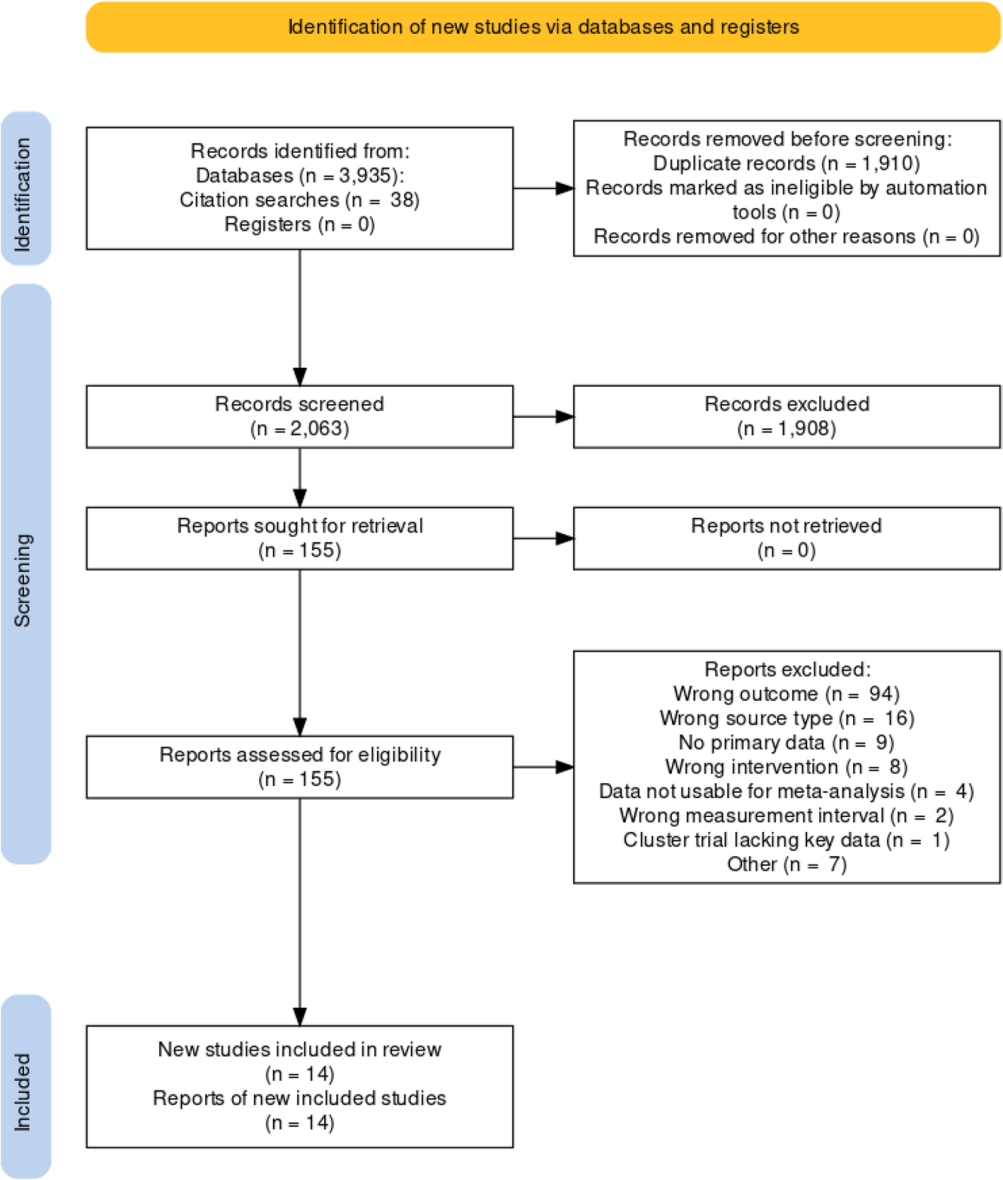
PRISMA flow diagram

Our database searches identified 3,935 records and our manual searches 38 records, of which 1,910 were duplicates. We screened the titles and abstracts of 2,063 studies, deeming 1,908 irrelevant. We screened the full texts of 155 studies, with Fig. 1 detailing reasons for exclusions. In the end, we included 14 studies. When screening abstracts and titles, Cohen’s kappa ranged from *κ* = 0.46 to *κ* = 1 across reviewer pairs; when screening full texts, it ranged from *κ* = 0.71 to *κ* = 0.81 across pairs.

Nine of the 14 included studies were NRSIs [31–39] and the rest RCTs [12, 40–43]. All nine NRSIs were uncontrolled trials. Coding was generally straightforward, though some data points such as percentage of females in the sample were sometimes missing in manuscripts. Together, studies included 286 participants with individual study sample sizes ranging from 4 to 42 participants (*x̄* = 20.43, *s* = 11.58, *x̃* = 20). Attrition rates ranged from 0 to 94% (*x̄* = 21.39%, *s* = 21.56%, *x̃* = 16.50%). Based on guidance, we classified 86% of these studies as feasibility studies, since they included fewer than 25 participants in total, or fewer than 25 participants per group [44].

Figure A, Figure B, and Figure C provide a tabular summary of included studies, but we also provide below a prose summary. Participants’ age ranged from 19 to 100 years (*x̄* = 75.45, *s* = 12.89, *x̃* = 77.55). Only two studies reported inclusion of participants younger than 50 years [12, 32]. A third study is likely to have included them [36]. Nevertheless, none of the three studies focused on participants younger than 50 years exclusively, and hence studies only included young participants along with older ones. Remaining studies explicitly reported excluding those younger than 50 [31, 33, 35, 37, 38, 40, 41, 43] or their sampling frames implied this [34, 39, 45]. Where reported, the percentages of both females and non-White participants were high in most studies.

Nine studies used social robotic relational agents [31–35, 39, 41, 43, 45] and five app-based relational agents [12, 36–38, 40]. The social robotic agents included Sony’s AIBO [32, 34, 41], PARO developed by ISRI [31, 33, 45], NAO developed by Aldebaran Robotics [35], Pepper developed by SoftBank Robotics [43], and either a robotic cat or dog developed by Joy for All [39]. The app-based agents included Laura developed by MIT [40], Elena + developed by ETH Zurich and the University of St. Gallen [36], Amazon’s Alexa [37], Bella by Soul Machines [46], and PACO developed by a consortium of Dutch organisations [38]. The relational behaviours of these agents varied. AIBO is a robotic puppy, PARO a robotic seal, and together with the robotic pets by Joy for All, these agents simulated live pet behaviour (e.g., the agents expressed emotions via facial cues and body language such as wagging of tails, played with users, learned their own names, and recognised users via their facial recognition capabilities) [32]. The agents responded to touch (e.g., petting) and adapted behaviour through reinforcement learning [33]. NAO and Pepper were humanoid robots that simulated human behaviours, customs, and speech. NAO, for example, would bow to users, extend its palm for a handshake, ask if participants would want to hear a poem, and only proceed once receiving a reply [35]. All app-based relational agents simulated humans. All were embodied, i.e., had a visual form, except for Amazon’s Alexa [37]. App-based agents primarily or to a significant degree used speech for relational behaviour. Laura, for example, expressed empathy (“I am sorry to hear that”), asked follow-up questions (“How tired are you feeling?”), and attempted to get to know users (“So, are you from the East Coast originally?”) [40]. Whilst in previous research several agents used “wizard-of-Oz methodologies”, i.e., agents controlled by humans pretending to be autonomous, all agents in this review were autonomous [47].

Most relational agents in our review acted as direct companions and did not seek to mitigate loneliness via other modalities [31–35, 37, 39–41, 43, 45], although exceptions existed. Elena + sought to remove cognitive biases and improve social skills [36]. PACO sought to create opportunities for socialising [38]. Bella sought to enhance social skills, increase social support, and increase opportunities for socialising [12].

Studies generally did not mention behavioural theories or behavioural change techniques (BCTs) that underpinned intervention design, although exceptions existed. One study based its intervention on Self-Determination Theory [38] and another study based its intervention on the COM-B model and the Theory of Planned Behaviour [36]. Nevertheless, these studies provided little detail on how exactly theories informed design. We classified BCTs according to the BCTTv1 by Michie et al., using below in quotation marks the labels of the original authors [48]. Only one study confirmed the full range of BCTs it used, and two other studies provided examples of BCTs. One study used six BCTs: “credible source”, “review behaviour goals”, “goal setting”, “instruction on how to perform a behaviour”, “social comparison”, and “social support” [38]. Another study mentioned seven BCTs: “information about emotional consequences”, “action planning”, “behavioural contract”, “instruction on how to perform a behaviour”, “review behaviour goals”, “reducing exposure to cues for the behaviour”, and “reduce negative emotions” [12]. A third study mentioned five BCTs: “information about emotional consequences”, “goal setting”, “instruction on how to perform a behaviour”, “reducing exposure to cues for the behaviour”, and “reduce negative emotions” [36].

**Fig. 2:**
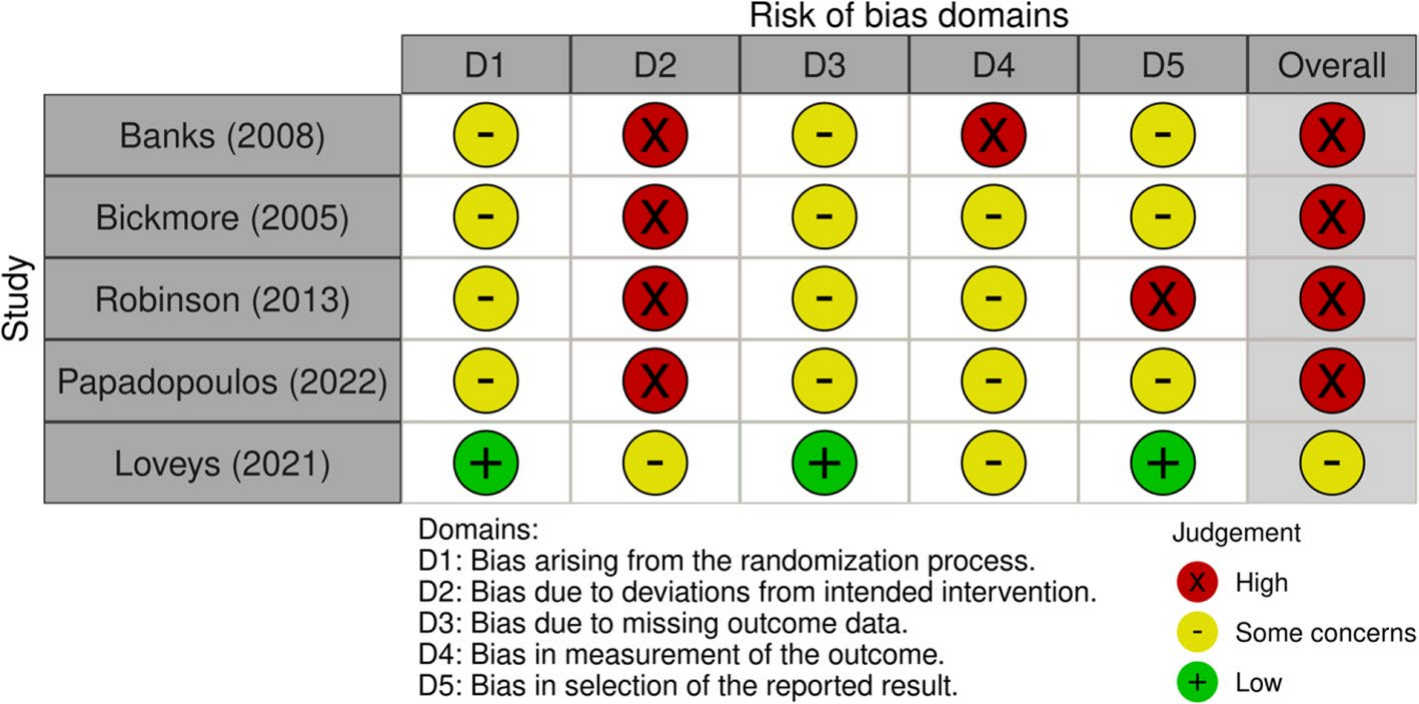
RCT risk of bias domains

**Fig. 3:**
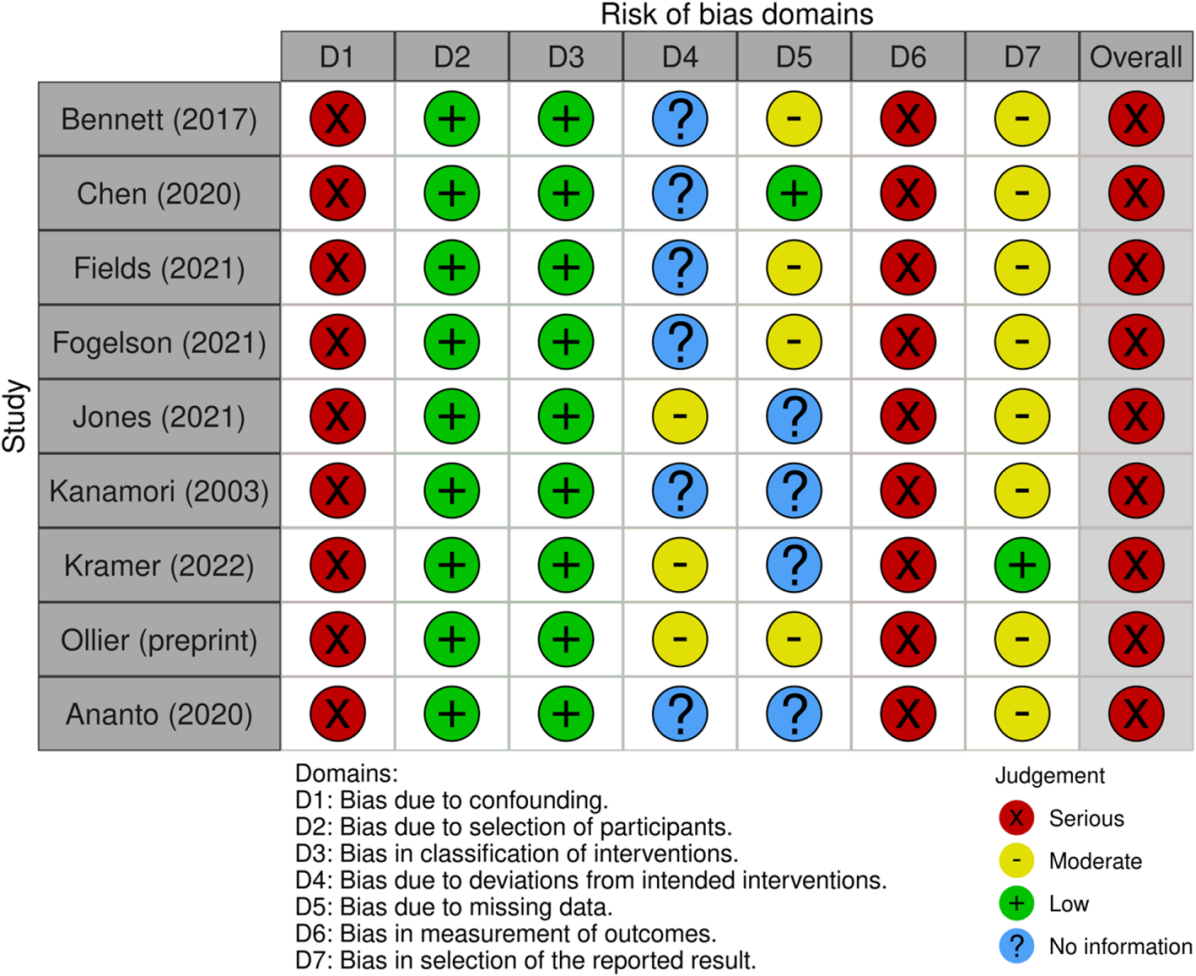
NRSI risk of bias domains

All RCTs were at high risk of bias due to potential deviations from the intended interventions [40, 41, 43, 45] except for one [12]. All NRSIs were at high risk due to confounds and potential biases in measurements [31–39]. Figures 2 and 3 illustrate these.

### Meta-analysis

**Fig. 4:**
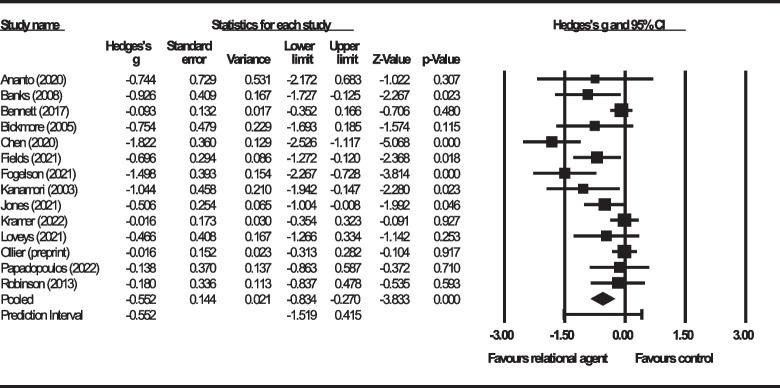
Main analysis forest plot

The pooled estimate of Hedge’s *g* was -0.552 (*Z* = -3.833; 95% CI, -0.834 to -0.270; *P* < 0.001), indicating on average a moderate effect of relational agents on loneliness reduction. This is shown in Fig. 4. Using a Bonferroni-corrected *α*_*Bonferroni*_ = 0.0125, there was evidence to reject the null hypothesis. Using the Knapp-Hartung adjustment, there was also evidence to reject the null hypothesis (*t* = -3.66; 95% Knapp-Hartung CI, -0.877 to -0.226; *P* = 0.003).

**Fig. 5:**
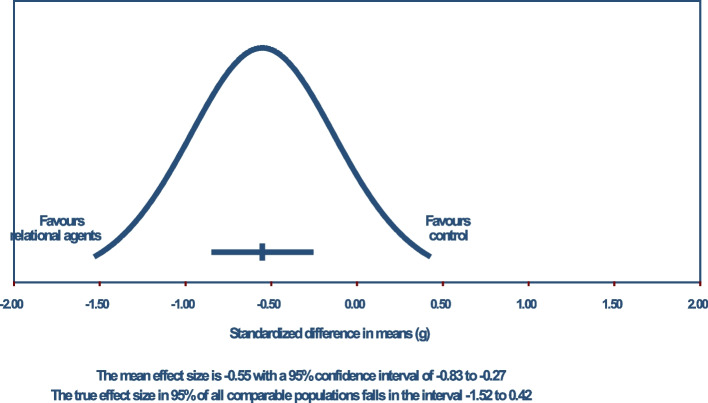
Main analysis prediction interval

Heterogeneity measures indicated that, as anticipated, the true effect of relational agents varied (*Q* = 45.073; *I*^*2*^ = 71%; *τ*^*2*^ = 0.176; *τ* = 0.420). Assuming a Gaussian distribution, the 95% prediction interval was estimated to range from -1.519 to 0.415, as seen in Fig. 5.

**Fig. 6:**
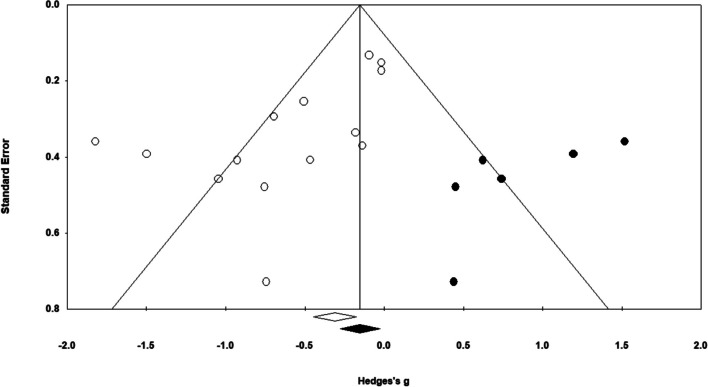
Funnel plot using standard error

Funnel plots as well as Egger’s test (*b* = -2.81; *t* = 3.5; *P* = 0.004) suggested that a small study effect may exist. Figure 6 illustrates this. The small study effect could have been due to effect sizes being larger in smaller studies or due to publication bias. Assuming a severe publication bias, the trim-and-fill algorithm resulted in an adjusted estimate of *g* = -0.198 (95% CI, -0.505 to 0.109), which attenuated the original estimate by roughly 64%.

Five studies were available for the RCT-only model. Hedge’s *g* was -0.437 (*Z* = -2.495; 95% CI, -0.781 to -0.094; *P* = 0.013), which was 21% less than the estimate of the main model. The results were significant at a traditional *α* = 0.05 but not at the *α*_*Bonferroni*_*.* The Knapp-Hartung adjusted results were not significant (*t* = -2.49; 95% Knapp-Hartung CI, -0.924 to 0.049).

Six studies were available for the app-based relational agent model. The pooled estimate of Hedge’s *g* was -0.286 (*Z* = -1.611; 95% CI, -0.553 to -0.020; *P* = 0.035), which was significant at a traditional *α* but not *α*_*Bonferroni*_. The Knapp-Hartung adjustment resulted in non-significant results (*t* = -2.11; 95% Knapp-Hartung CI, -0.636 to 0.063). Eight studies were available for the social robotic relational agent model. The pooled estimate of Hedge’s *g* was -0.774, which was significant at *α*_*Bonferroni*_ (*Z* = -2.909; 95% CI, -1.296 to -0.252; *P* = 0.004). Using a Knapp-Hartung adjustment, results were significant at a traditional *α* but not at *α*_*Bonferroni*_ (*t* = -2.91; 95% Knapp-Hartung CI, -1.403, -0.145, *P* = 0.023).

## Discussion

Our review is the first to provide quantitative evidence for the efficacy of relational agents to reduce loneliness in participants aged 19 to 100 years. Our results are promising, and although the effect size of *g* = -0.552 is likely somewhat inflated due to publication bias, it is probably less inflated than our trim-and-fill algorithm suggested. This is because the trim-and-fill algorithm assumed that several studies were suppressed in which relational agent interventions exacerbated loneliness. This, however, is unlikely. Failed loneliness interventions tend to have no effect on loneliness, not exacerbate it [10]. Our review could have used different algorithms to adjust for publication bias, and alternatives would probably have yielded different adjustments. Recently, for example, researchers have applied four different algorithms to a high-profile meta-analysis, resulting in a mix of significant and non-significant adjustments [49–51]. Nevertheless, no algorithm for publication bias would provide the “correct” effect size [52]. Instead, algorithms provide a sensitivity analysis assuming certain parameters, and sometimes these parameters lead to flawed results, e.g., the trim-and-fill algorithm overcorrects under heterogeneity, which was the very assumption of our analysis [53]. Ultimately, the most likely interpretation is that the true average effect size of relational agents was small to moderate. Table 1 provides a summary of our results.

**Table 1:** Summary of results

	**Main analysis**	**Main analysis with Knapp-Hartung adjustment**	**Trim-and-fill analysis**	**RCT-only analysis**	**App-only analysis**	**Social robotic-only**
Hedge’s *g*	-0.552	-0.552	-0.198	-0.437	-0.286	-0.774
95% CI	-0.834 to -0.270	-0.877 to -0.226	-0.505 to 0.109	-0.781 to -0.094	-0.553 to -0.020	-1.296 to -0.252
*P*	< 0.001	0.003	Not significant	0.013	0.035	0.004

We believe the above results have two important implications for the current loneliness literature. First, the literature is in search for novel and effective interventions that are scalable. The NHS is already facing resource constraints, these constraints are expected to exacerbate, and the NHS has consequently called for the increased adoption of AI to ease its burden [54]. Relational agents can be highly scalable, once some groundwork has been completed, and a possible follow-up from our results is a national or regional pilot. Such a pilot, of course, would entail the resolution of complex issues (e.g., digital literacy, access to technology, and privacy). Researchers, for example, will need to determine who will have access to user data and in what form, and such choices can fundamentally impact the success of a pilot.

The second implication of our results is that relational agents may act as a standalone intervention, but they are likely to be more useful in multi-component interventions that are tailored to individual needs. In the UK, the NHS’s current main strategy for loneliness is “social prescribing”, an outsourcing approach in which staff refer individuals to community schemes such as lifestyle interventions (e.g., physical exercise) or social activity interventions (e.g., volunteering) [7, 55]. While there are alternative intervention approaches for loneliness, social prescribing is viewed by individuals and service providers as helpful [7] and cost-effective [56, 57]. Social prescribing is, in essence, a sign-posting intervention, and it could sign-post, among other things, to relational agents. This could be valuable because there is currently a notion that interventions improve lives, but that people do not recover from loneliness [58]. Potentially, this may be because not all loneliness is the same. Two people may feel lonely for two different reasons, and these people may then require different sets of solutions [58]. Relational agents can extend the set of available solutions, and agents can complement existing human-centred interventions, rather than replace them.

Relational agents, thus, could help in the fight against loneliness. What is more, their full potential has not yet been realised. On the one hand, this is due to the absence of state-of-the-art knowledge integration. For example, the use of behavioural theories and BCTs can enhance intervention efficacy, yet studies in our sample generally did not discuss such theories and BCTs. Similarly, interventions can modify loneliness via multiple modalities. Studies in our review, however, generally used only one of these modalities, and the others—such as the debiasing of social cognition that has shown particular promise [56]—are yet to be integrated into relational agent design [14]. On the other hand, relational agents have not yet realised their full potential due to the nascency of AI. Increasingly, LLMs are powering relational agents. These models allow relational agents to produce open-ended, original, and highly tailored conversation, and although much of the conversation of relational agents has already become indistinguishable from human conversation [59], research on LLMs is burgeoning, and the race is on between organisations such as OpenAI and Google to develop the next generation of LLMs [60].

### Limitations

Our review faced common limitations such as the exclusion of non-English sources and the quality of underlying primary studies, but a particular limitation of our review were the mixed results of the sensitivity and sub-group analyses. There are three potential explanations for this. First, sample sizes in these sub-group analyses were less than 10, and analyses with fewer than 10 studies tend to lack power [52]. At the same time, it is likely that underlying studies themselves lacked power due to small sample sizes [61]. Indeed, Appendix C demonstrates that power was likely well below the recommended level of 80% in our sub-group analyses, while Appendix D presents an additional sensitivity analysis indicating that further primary studies would have meaningfully reduced *p* levels [52]. Second, our review may have tested for results too conservatively. The Bonferroni correction, as applied in this review, results in Type 2 error rates of roughly 33%, which some have referred to as unacceptably high [62]. Finally, our review conducted two-tailed significance tests. This is usually anodyne—since interventions can both improve and exacerbate outcomes. Nevertheless, in cases where interventions are unlikely to exacerbate outcomes, one-tailed tests may be warranted [52]. This, as discussed, is likely to be the case with loneliness and relational agents. Had we conducted one-tailed tests, this would have entailed the halving of *p* values, which would have made some results statistically significant. Third, execution may have been a problem. Primary studies may not have sufficiently exposed participants to relational agents, or participants may not have interacted with relational agents, or relational agents may not have been correctly designed. Chen et al. [63], for example, found no significant difference between control and experimental groups at a four-week interval [63]. They did, however, find a significant difference at an eight-week interval. In our review, the mean time between pre-test and final post-test was 5.92 weeks.

### Future research

We lack an understanding of relational agents in several areas, and we suggest that future research could focus on three. First, research on relational agents and loneliness in young people is scarce. Among some youth groups loneliness rates are higher than those of the elderly, and these rates of youth loneliness are increasing [64]. At the same time, smartphone ownership is high among the young [64]. Young people therefore are pertinent and amenable for the study of loneliness. Second, the efficacy of relational agents will depend on a variety of population and design factors. On the population side, we suspect that factors such as age, education, and digital literacy may impact efficacy. On the design side, we suspect that a hierarchy of features exists, e.g., certain design features will deliver more bang for your buck, although it is less clear which [65]. Third, although general attitudes towards relational agents may be favourable, some are concerned about the introduction of relational agents and similar technologies [66]. Future research could therefore explore how technology should be harnessed to increase its benefits and reduce unintended consequences. Finally, future research could address the shortcomings of current research. Almost all underlying studies in our review suffered from high risk of bias in one or several domains, sample sizes were small, and follow-up periods were brief. Particularly, there is a need for more high-quality RCTs.

## Conclusion

The current study is the first meta-analysis to explore the effects of relational agents on loneliness across all age groups. It is also the first meta-analysis to provide statistically significant evidence for the efficacy of relational agents, which on average had a moderate effect on loneliness reduction. Loneliness has serious physical and mental health consequences for individuals, and the monetary costs to the state and employers are staggering [67–69]. Unfortunately, current interventions for loneliness can suffer from low engagement and scalability [58]. Relational agents, on the other hand, are an emerging technology that due to advances in AI and LLMs will increase in sophistication and realism. Although a multi-pronged approach is required, relational agents could play a significant role in alleviating a growing public health concern [64]. Future work is required that addresses weaknesses of current studies such as risk of bias, small study size, and brief follow-up periods.

### Supplementary Information

Supplementary Material 1.

Supplementary Material 2.

## Data Availability

As a meta-analysis, this study used data reported in the literature. Appendices and derived data for meta-analytic calculations are available the Open Science Framework here https://osf.io/c6rdk/files/osfstorage. Reader can also write directly to the corresponding author.
