# Supplementary material for: Efficacy of relational agents for loneliness across age groups: a systematic review and meta-analysis
Source: BMC Public Health. 2024 Jul 6;24:1802. doi: 10.1186/s12889-024-19153-x (PMC11227208; doi:10.1186/s12889-024-19153-x)
Supplement: Supplementary file 1 — Supplementary Material 1. [file 12889_2024_19153_MOESM1_ESM.docx]

# APPENDIX A: FULL SEARCH STRATEGY

The full search strategy for each database can be found in a spreadsheet (.xlsx format) hosted on the Open Science Framework’s website: <https://osf.io/eyqjx>.

# APPENDIX B: DATA IMPUTATIONS

A spreadsheet can be found here, which explains what data imputation decisions we made: <https://osf.io/hynt5>.

# APPENDIX C: STATISTICAL POWER

The power of a hypothesis test is the probability that the null hypothesis is rejected when false ^1^. Power analysis for primary studies use formulas to evaluate a parameter *λ* against a standard normal distribution *Φ(|Z|)*, and these formulas can be adapted for meta-analyses ^1^. Here, the study presents a simplified, apriori calculation conducted before any data were collected and that used RCTs assuming two independent groups as a basis of its calculation. These calculations are conservative, draw on the results by Gasteiger et al.^2^, the review most comparable to ours, and the calculations assume the following.

- *α* = 0.05,
- a main effect size of *d* = 0.4 (which is half the median reported effect size in Gasteiger et al. which was *d* = 0.8),
- the existence of *k* = 10 experiments (Gasteiger et al.^2^ identified 8),
- group sizes of *n* = 14 (which is the median group size in Gasteiger et al.^2^), and
- moderate between-studies variance of *𝜏^2^* = 1.667.

The within-study variance for one study is calculated using

| $V_{d}=\frac{n_{1}+ n_{2}}{n_{1}\times n_{2}}+ \frac{d^{2}}{2(n_{1}\times n_{2})}$. | (1.1) |
| --- | --- |

The variance of the summary effect is computed as

| $V_{\delta}=\frac{V_{Y} + \tau^{2}}{k}$. | (1.2) |
| --- | --- |

The parameter *λ* is given by

| $\lambda=\frac{\delta}{\sqrt{V_{\delta}}}$. | (1.3) |
| --- | --- |

Finally, the power of a study is found via

| $Power=\left( 1- \Phi\left( c_{\alpha}- \lambda\right) \right)+ \Phi(-c_{\alpha}- \lambda)$. | (1.4) |
| --- | --- |

Under these assumptions, our meta-analysis will have a power of 74% to detect a main effect size of 0.4. This is close to the recommended power of 80%, and it exceeds the power of a single primary study^3^. Under the above assumptions, a single primary study would have a power of only 18% to detect a main effect of the same size.

When the same above calculation is repeated assuming 5, 6, and 8 studies, instead of 10 studies, the power of the meta-analysis changes. Under the above assumptions, such meta-analyses will have a power of 44%, 51%, and 63% respectively. This suggests that although some of the sub-group and sensitivity analyses in this study were approaching the recommended power of 80%, these analyses as a group were underpowered due to the scarcity of studies^3^.

# APPENDIX D: ADDITIONAL SENSITIVITY ANALYSIS

The study conducted an additional sensitivity analysis to explore the impact of including one further study in the RCT-only meta-analysis. Here, a study by Chen et al.^4^ was included that was excluded from the main body of this study due to lacking too many essential data points, e.g. intra-cluster correlation coefficient (ICC) ^4^. In this sensitivity analysis, the study assumed an *F*-value of 7.16, provided by Chen et al.^4^, the number of floors in the control and treated group to be 4 respectively, the number of patients on the floor to be 6.5, and an ICC of 0.01. The estimate of the ICC was based on a study by Adams et al.^5^ and the cluster sizes seemed reasonable based on the total reported sample size in Chen et al. Without the Chen et al. study, the RCT-only model in the main body was not significant with a Knapp-Hartung adjustment, but when the study was included, the model became significant at a traditional *α* (*t* = -3.48; *df* = 5; 95% adjusted CI, -0.902 to -0.136; *P* = 0.018, two tailed). The attenuation of Hedge’s *g* was reduced to -0.519, which was only 6% less in magnitude than the estimate of *g* in the main analysis.

# APPENDIX E: RELATIONAL AGENTS

A video link for Sony’s AIBO (social robotic) is [here](https://www.youtube.com/watch?v=J4AvPo5P_Fg) and a link for Luka’s Replika (app-based) is [here](https://www.youtube.com/watch?v=yQGqMVuAk04).

# APPENDIX F: SCREENING AND DATA EXTRACTION FORMS

All forms are hosted on the Open Science Framework’s website: https://osf.io/cukyp and <https://osf.io/5n96e>.

# APPENDIX G: RISK OF BIAS ASSESSMENT

**RCTs**

The RoB 2 classifies bias in five domains as “low”, “some concerns”, or “high” based on multiple-choice questions. These five domains are: bias arising from the randomisation process, bias due to deviations from intended interventions, bias due to missing outcome data, bias in measurements of the outcomes, and bias in selection of the reported results. An algorithm then determines the classification of bias in each domain and for studies as a whole^6^.

RCTs generally raised some concerns regarding the randomisation process since studies lacked descriptions of the exact randomisation mechanisms. A high risk of bias existed due to deviations from the intended interventions, as studies did not or could not control for non-adherence or the existence of unintended interventions. There were some concerns regarding bias due to missing outcome data, given attrition rates in included studies. There were some concerns when assessing bias in the measurement of the outcome due to participants being aware of their experimental condition. Some concerns regarding the reporting of results existed due to the absence of pre-registered protocols. Given the preponderance of high-risk of bias due to deviations from intended interventions, the RoB 2 algorithm deemed most RCTs at high overall risk of bias.

*Figure 1: RCT risk of bias summary*

*
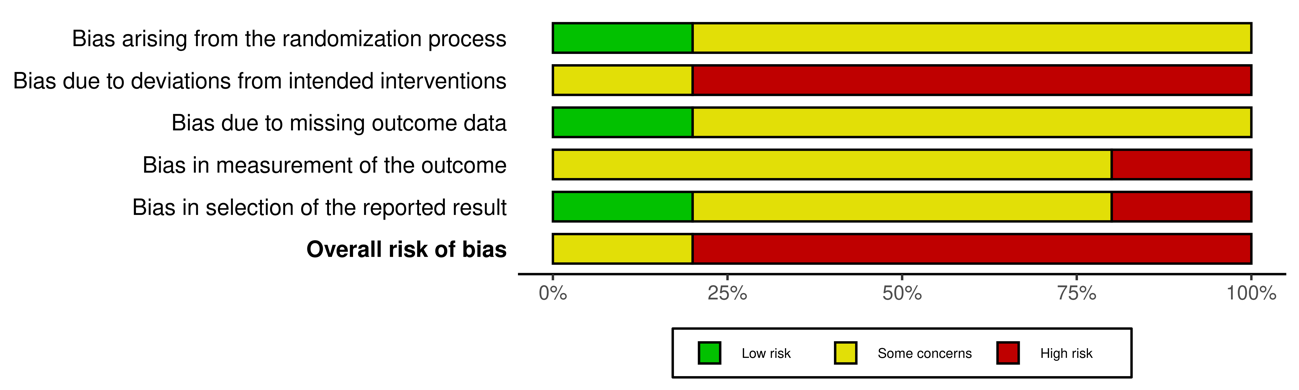
*

**NRSIs**

The ROBINS-I is based on the RoB 2 so shares most of the latter’s features. The difference is that the ROBINS-I algorithm is less formulaic, that the tool labels “some concerns” as “moderate”, and that two additional domains of bias are assessed: bias due to confounding and bias due to selection of participants^7^.

All included studies exhibited a high risk of bias due to confounding since studies could not control for expectancy effects. Risk of bias to due participant selection was low as the domain is primarily concerned with post-intervention selection biases, which did not apply to included studies. Similarly, risk of bias due to the classification of interventions was low since studies clearly distinguished between participants that did and did not receive the intervention. Many included studies lacked data to assess the risk due to deviations from intended intervention as well as bias due to missing data. Since participants were aware of their experimental group, a high risk of bias existed for most studies in the measurement of outcomes. The risk of bias in selection of the reported results was moderate in most cases due to the absence of pre-registered protocols. Given that included NRSIs could not and did not control confounders, the ROBINS-I algorithm deemed all studies at high risk of bias overall.

*Figure 9: NRSI risk of bias summary*


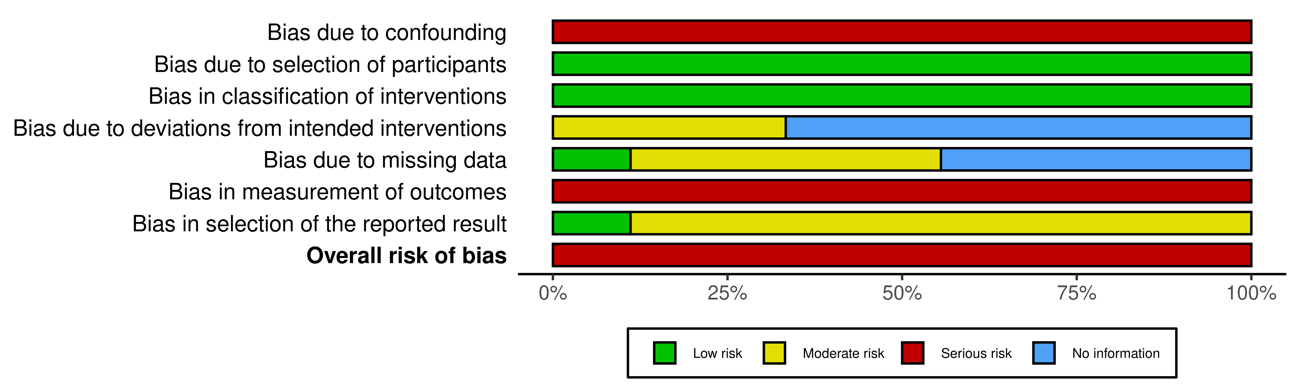


**APPENDIX H: RISK OF BIAS ASSESSOR SHEETS**

Assessor sheets are available here: <https://osf.io/c6rdk/files/osfstorage>.

**APPENDIX I: COMPUTATIONAL DATA**

Data used in our meta-analytic computations are available here: <https://osf.io/hbacv>.

**APPENDIX J: EXCLUDED STUDIES AND REASONS**

A list of excluded studies and reasons for exclusions can be found here: <https://osf.io/avdjg>.

**REFERENCES ACROSS ALL APPENDICES**

1. Borenstein M Hedges, Larry V, Higgins J Rothstein, Hannah R. *Introduction to Meta-Analysis*. Wiley; 2021.

2. Gasteiger N, Loveys K, Law M, Broadbent E. Friends from the Future: A Scoping Review of Research into Robots and Computer Agents to Combat Loneliness in Older People. *Clin Interv Aging*. 2021;16:941-971. doi:10.2147/CIA.S282709

3. Cohen J. A power primer. *Psychol Bull*. 1992;112(1):155-159. doi:10.1037//0033-2909.112.1.155

4. Chen SC, Jones C, Moyle W. The Impact of Engagement with the PARO Therapeutic Robot on the Psychological Benefits of Older Adults with Dementia. *Clin Gerontol*. 2022;0(0):1-13. doi:10.1080/07317115.2022.2117674

5. Adams G, Gulliford MC, Ukoumunne OC, Eldridge S, Chinn S, Campbell MJ. Patterns of intra-cluster correlation from primary care research to inform study design and analysis. *J Clin Epidemiol*. 2004;57(8):785-794. doi:10.1016/j.jclinepi.2003.12.013

6. Sterne J, Savović J, Page MJ, et al. RoB 2: a revised tool for assessing risk of bias in randomised trials. *BMJ*. 2019;366:l4898. doi:10.1136/bmj.l4898

7. Sterne J, Hernán MA, Reeves BC, et al. ROBINS-I: a tool for assessing risk of bias in non-randomised studies of interventions. *BMJ*. 2016;355:i4919. doi:10.1136/bmj.i4919
